# Supplementary material for: The interactome of intact mitochondria by cross-linking mass spectrometry provides evidence for coexisting respiratory supercomplexes
Source: Mol Cell Proteomics. 2017 Dec 8;17(2):216–32. doi: 10.1074/mcp.RA117.000470 (PMC5795388; doi:10.1074/mcp.RA117.000470)
Supplement: Supplemental Data [file supp_RA117.000470_133922_0_supp_23978_fzffwf.zip › spectra_annotation/mito_spectra_annotation/386-1-12-1-10-1.pdf]

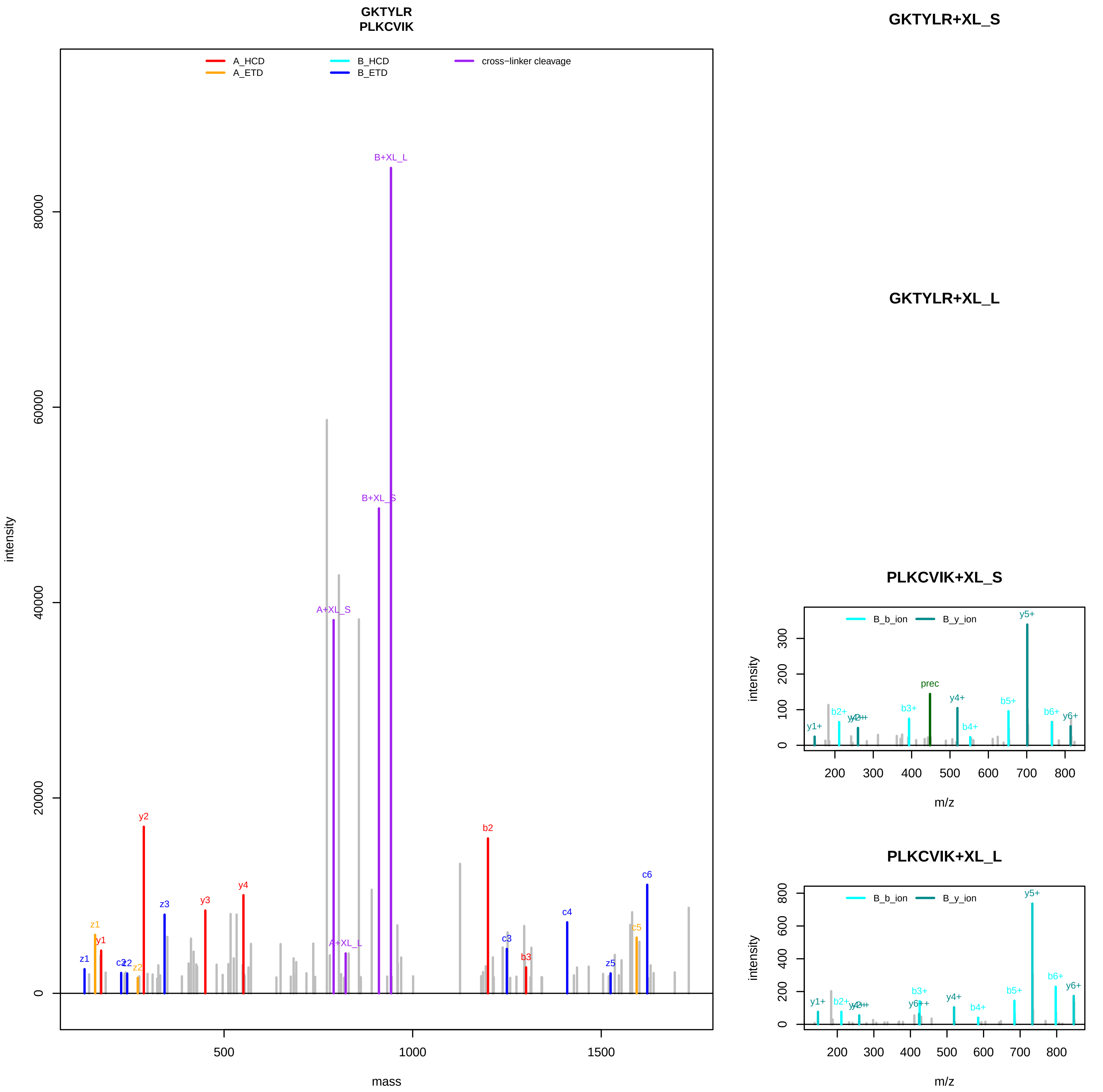
GKTYLR
PLKCVIK
A_HCD
A_ETD
B_HCD
B_ETD
cross−linker cleavage
B+XL_L
B+XL_S
A+XL_S
A+XL_L
z1
y1
y2
z3
y3
y4
b2
c3
b3
c4
z5
c5
c6
intensity
mass
0
20000
40000
60000
80000
500
1000
1500
GKTYLR+XL_S
GKTYLR+XL_L
PLKCVIK+XL_S
B_b_ion
B_y_ion
y1+
b2+
b3+
prec
y4+
b4+
b5+
y5+
b6+
y6+
m/z
PLKCVIK+XL_L
0
100
200
300
400
500
600
700
800
